# Microbiome and chronic pelvic pain in women: a mini-review

**DOI:** 10.1093/humrep/deag110

**Published:** 2026-07-24

**Authors:** Jonas Vibert, Milos Stojanov, Tatiana Da Silva, David Baud, Nicola Pluchino

**Affiliations:** Reproductive Medicine Unit (UMR), Service of Gynecology, Department Woman-Mother-Child, Lausanne University Hospital (CHUV) and University of Lausanne, Lausanne, Switzerland; Materno-Fetal and Obstetrics Research Unit, Department Woman-Mother-Child, Lausanne University Hospital and University of Lausanne, Lausanne, Switzerland; Materno-Fetal and Obstetrics Research Unit, Department Woman-Mother-Child, Lausanne University Hospital and University of Lausanne, Lausanne, Switzerland; Materno-Fetal and Obstetrics Research Unit, Department Woman-Mother-Child, Lausanne University Hospital and University of Lausanne, Lausanne, Switzerland; Reproductive Medicine Unit (UMR), Service of Gynecology, Department Woman-Mother-Child, Lausanne University Hospital (CHUV) and University of Lausanne, Lausanne, Switzerland

**Keywords:** chronic pelvic pain, microbiota, dysbiosis, endometriosis, estrobolome, gastrointestinal microbiome, vagina/microbiology, irritable bowel syndrome, neuroinflammation, probiotics

## Abstract

Chronic pelvic pain (CPP) is a prevalent, disabling syndrome encompassing overlapping disorders such as endometriosis/adenomyosis, bladder pain syndrome/interstitial cystitis, irritable bowel syndrome, vulvodynia, and myofascial pain syndrome. Despite distinct clinical phenotypes, these conditions converge on shared biological axes—immune dysregulation, endocrine imbalance, and central sensitization—that sustain chronic pain. Increasing evidence implicates the human microbiome as a potential upstream regulator of these pathways. Dysbiosis across the gut, vaginal, urinary, and endometrial microbial ecosystems may promote local and systemic inflammation, compromise epithelial barrier integrity, alter estrogen recirculation through the estrobolome, and engage aberrant neuroimmune signalling along gut–brain and hypothalamic–pituitary–ovarian circuits. Recent multi-site profiling suggests that microbial alterations often co-occur across pelvic compartments but remain anatomically distinct, with shifts in anaerobic taxa and paired cervicovaginal immune signatures supporting microbiome–immune interactions in CPP pathophysiology. This narrative review synthesizes observational, multi-omics, and mechanistic evidence linking microbial dysbiosis to CPP, highlights microbial metabolites as key functional mediators, and evaluates causal data from experimental models. Finally, it discusses translational opportunities and limitations, including microbiome-targeted interventions (dietary modulation, probiotics/psychobiotics, postbiotics, and microbiota transfer approaches) and the need for harmonized, longitudinal and biomarker-embedded trials to enable mechanism-based stratification and rational therapeutic development.

## Graphical Abstract

**Graphical Abstract:**
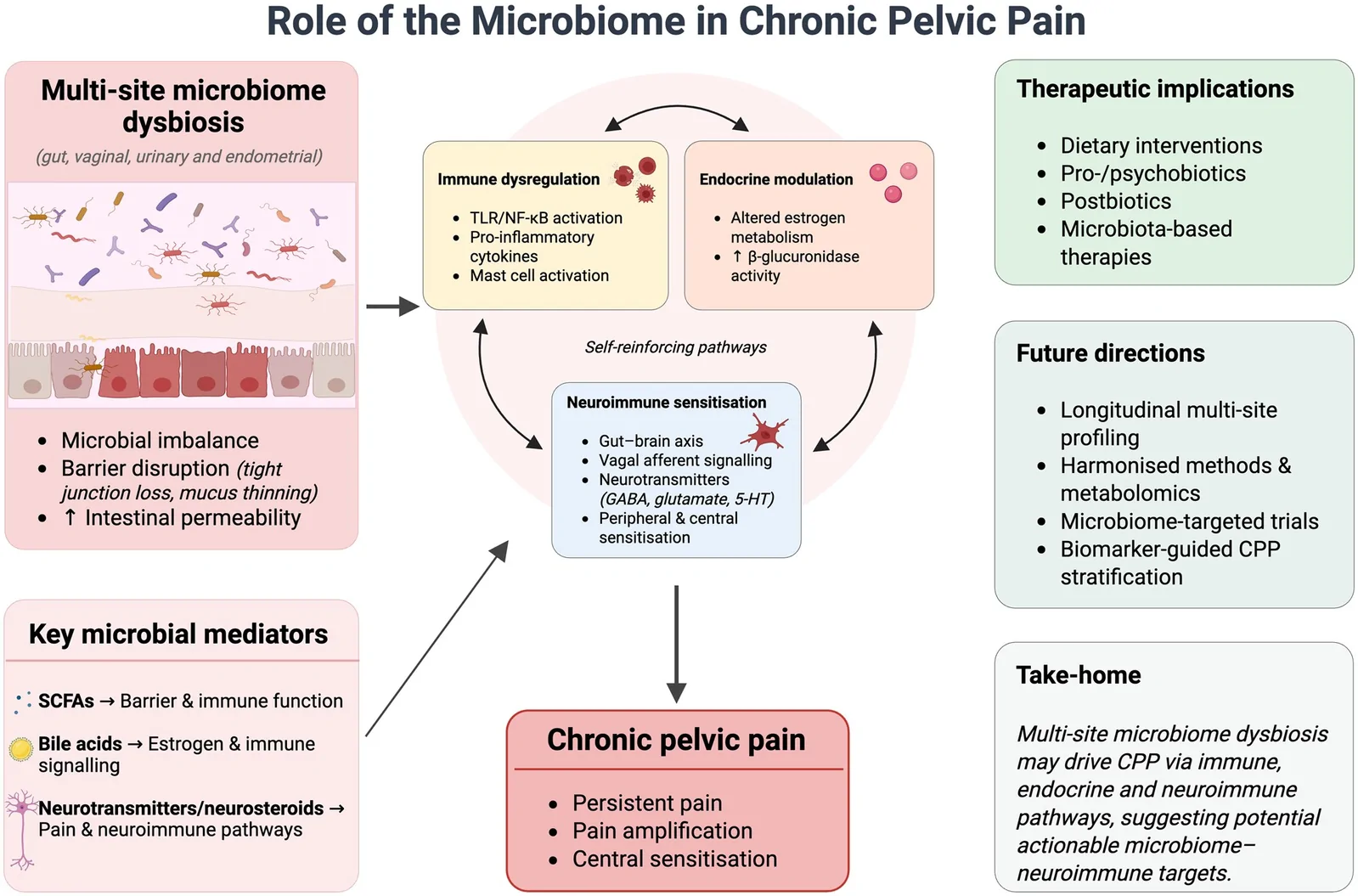
Role of the microbiome in chronic pelvic pain. 5-HT, 5-hydroxytryptamine; CPP, chronic pelvic pain; GABA, gamma-aminobutyric acid; SCFA, short-chain fatty acids; TLR, Toll-like receptor.

## Introduction

Chronic pelvic pain (CPP) is defined as noncyclic pain perceived in pelvic structures lasting at least 6 months and severe enough to cause functional disability or psychosocial distress (Chronic Pelvic Pain, 2020; Lamvu *et al.*, 2021). It affects ∼5.7–26.6% of women of reproductive age worldwide, depending on the diagnostic criteria used and represents one of the leading indications for laparoscopy (∼40% of cases) and hysterectomy (∼12% of cases), most of which are probably unnecessary (Chronic Pelvic Pain, 2020; Lamvu *et al.*, 2021).

CPP often encompasses overlapping conditions such as endometriosis/adenomyosis, bladder-pain syndrome/interstitial cystitis (BPS/IC), irritable bowel syndrome (IBS), vulvodynia, and myofascial pain syndrome (Chronic Pelvic Pain, 2020; Lamvu *et al.*, 2021). Despite their distinct clinical phenotypes, these syndromes converge on three interrelated biological axes that together sustain chronic pain: immune dysregulation, endocrine imbalance, and central sensitization (Salliss *et al.*, 2021; Karp and Stratton, 2023; Cuffaro *et al.*, 2024; Cardaillac *et al.*, 2025).

**Figure 1.**
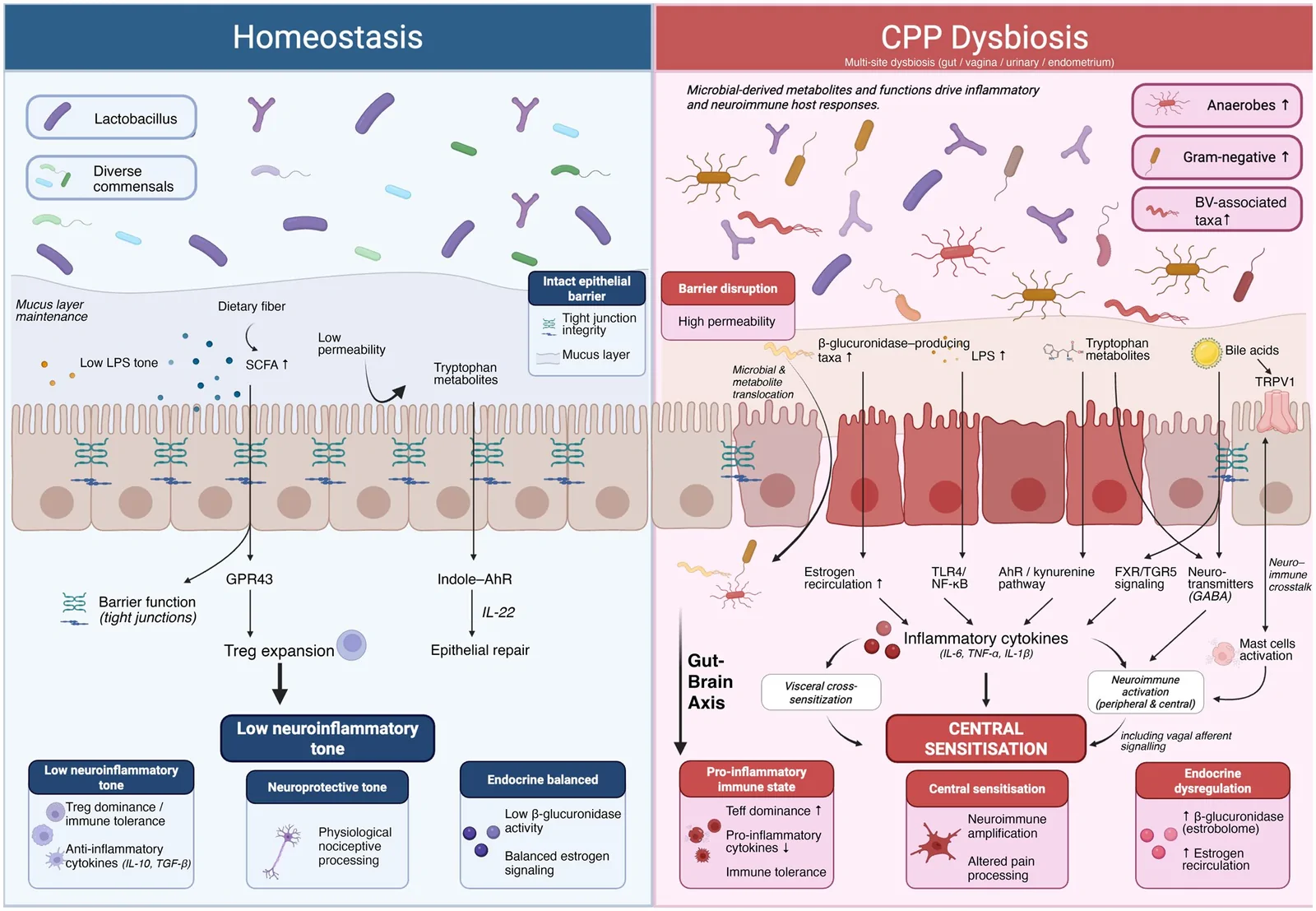
**Microbiome-driven mechanisms linking epithelial barrier function, immune-endocrine signalling and central sensitization in chronic pelvic pain (CPP)**. This schematic illustrates a systems-level model linking microbial homeostasis (left) to multi-site dysbiosis and CPP pathophysiology (right) across the gut, vaginal, urinary, and endometrial compartments. **Homeostasis (left)**: Lactobacillus-dominated and diverse commensal communities maintain mucus integrity and tight junctions, resulting in low epithelial permeability and low LPS tone. Short-chain fatty acids (SCFAs) derived from dietary fibre signal via GPR43 to promote regulatory T cell (Treg) expansion and immune tolerance. Microbial tryptophan metabolites activate the aryl hydrocarbon receptor (AhR), supporting epithelial repair and barrier protection (IL-22 pathways). Low β-glucuronidase activity preserves balanced estrogen signalling. Together, these mechanisms sustain endocrine balance, low neuroinflammatory tone, and physiological nociceptive processing. **CPP dysbiosis (right)**: Enrichment of anaerobic, Gram-negative, and BV-associated taxa is associated with barrier disruption and increased epithelial permeability. Elevated LPS activates TLR4/NF-κB signalling and pro-inflammatory cytokine production. Increased β-glucuronidase activity enhances estrogen recirculation, contributing to endocrine dysregulation. Altered bile acid and tryptophan metabolism engage FXR/TGR5 and AhR/kynurenine pathways, while TRPV1 activation contributes to peripheral sensitization. These converging immune, endocrine, and metabolic signals propagate along the gut–brain axis, including vagal afferent signalling and microbiota-derived neurotransmitters such as GABA, promoting neuroimmune activation and central sensitization, resulting in a pro-inflammatory state and persistent CPP. AhR, aryl hydrocarbon receptor; BV, bacterial vaginosis; FXR, farnesoid X receptor; GABA, gamma-aminobutyric acid; GPR43, free fatty acid receptor 2; IL, interleukin; LPS, lipopolysaccharide; SCFA, short-chain fatty acids; TGR5, Takeda G-protein-coupled receptor 5; TLR4, Toll-like receptor 4; Treg, regulatory T cell; TRPV1, transient receptor potential vanilloid 1.

In recent years, the human microbiome has emerged as a potential *upstream regulator* of these pathways. Dysbiosis across the gut, vaginal, urinary, and endometrial microbiota may promote local and systemic inflammation, disrupt epithelial–barrier integrity, alter estrogen metabolism via the estrobolome and activate aberrant neuro-immune signalling along the gut–brain and hypothalamic–pituitary–ovarian axes (Rahman-Enyart *et al.*, 2021; Cuffaro *et al.*, 2024; Hearn-Yeates *et al.*, 2024; Li *et al.*, 2025; Ren *et al.*, 2025). Recent studies revealed that dysbiosis rarely occurs in isolation within a single pelvic compartment (Jimenez *et al.*, 2024). A systems-level overview of these interactions is presented in Fig. 1.

This narrative review synthesizes current evidence linking bacterial, viral, and fungal dysbiosis to CPP, highlighting mechanistic pathways, clinical implications, and emerging microbiome-targeted therapeutic strategies. The literature search strategy is detailed in Supplementary Materials and Methods.

## Current evidence

### Microbial ecosystem disruption across pelvic sites

#### Lower and upper reproductive tract microbiota

The pelvic microbiota forms an interconnected ecological network and disruptions within these niches are associated with CPP (Salliss *et al.*, 2021). In the vagina, *Lactobacillus* species dominate and maintain epithelial homeostasis through acidification (France *et al.*, 2022). Dysbiosis, marked by reduced *Lactobacillus* spp. and overgrowth of *Prevotella*, *Gardnerella*, and *Atopobium*, is repeatedly linked to increased pain intensity, elevated interleukin-8 (IL-8) concentrations, and a heightened inflammatory milieu among women with endometriosis or severe dysmenorrhea (Salliss *et al.*, 2021; Jimenez *et al.*, 2024). Additional alterations in endometriosis- and adenomyosis-related CPP include increased *Clostridium butyricum*, *Clostridium disporicum*, *Alloscardovia omnicolens*, and *Veillonella* sp. (Chao *et al.*, 2021), suggesting a more complex vaginal signature in hormonally responsive pelvic pain disorders.

The endometrium harbours a low-biomass microbial community, whose composition and physiological role is still debated (Pelzer *et al.*, 2018; Wessels *et al.*, 2021; Reschini *et al.*, 2022). Although evidence supports the concept that the endometrial microbiome is biologically relevant to endometriosis, no robust, reproducible disease-specific microbial signature has been identified, with associations remaining inconsistent across studies (Facciotti *et al.*, 2025). This likely reflects methodological and biological confounding rather than absence of a microbiome-disease link. Small and underpowered cohorts, inadequate control for menstrual cycle phase, hormonal treatments, diet, and geography, contamination in low-biomass samples, heterogeneous sequencing platforms, inconsistent bioinformatic pipelines, and differences between central or commercial analytical platforms undermine comparability and reproducibility (Gajer *et al.*, 2012; Yatsunenko *et al.*, 2012; Brooks *et al.*, 2018; Knight *et al.*, 2018; Fierer *et al.*, 2025). Findings from studies relying on non-standardized analytical approaches should therefore be interpreted with appropriate caution.

Reported enrichments in endometrial samples include *Streptococcus*, *Gardnerella*, and *Prevotella* genera, but vary across studies. Given the low bacterial biomass and contamination risk, these genus-level signals remain fragile. Genus-level taxonomic resolution is therefore insufficient to infer causality or mechanistic relevance. Many genera reported as differentially abundant encompass both commensal and potentially pathogenic species, as well as strains with profoundly divergent metabolic and immunomodulatory capacities. As a result, taxonomic associations at the genus level frequently obscure functionally relevant variation and may explain the poor reproducibility observed across studies. The case of *Fusobacterium nucleatum* is illustrative in this regard. Muraoka *et al.* reported its enrichment in the endometrial cavity of women with endometriosis, with antibiotic treatment reducing lesion burden in a murine model (Muraoka *et al.*, 2023). However, these findings have not been consistently reproduced, highlighting the difficulty of validating low-biomass endometrial signatures.

Key studies examining vaginal and endometrial microbiota in relation to CPP are summarized in Table 1.

**Table 1.** Lower and upper female reproductive tract (FRT) microbiome studies.

Reference	Study design	N/Model	CPP condition	Key microbial findings	Pain/clinical outcome
Jimenez *et al.* (2024)	Cross-sectional observational	Pilot clinical cohort (n=73)	CPP ± endometriosis	Vaginal & rectal; ↑ Prevotella spp., ↑ vaginal Streptococcus anginosus in CPP-Endo vs CPP alone, rectal dysbiosis including IBS-associated taxa, and BV-associated taxa mainly in AUB/fibroid subgroup analyses; cervicovaginal immune alterations including reduced MDC, IL-8, VEGF and GRO (CXCL1)	Pain not directly scored; cervicovaginal immune signatures correlated with CPP phenotype
Salliss *et al.* (2021)	Systematic review	28 clinical + 6 animal studies	Endometriosis, CPP	Cervicovaginal: BV-associated bacteria enriched; Lactobacillus depleted; no clear consensus on specific taxa; endometrial data limited by methodological heterogeneity. Animal studies support a bidirectional relationship between the gut microbiota and endometriosis onset and progression	Putative microbiome–CPP association via gut-brain axis; no direct pain–microbiome studies
Chao *et al.* (2021)	Observational	Clinical cohort (n=128)	Endometriosis/adenomyosis	Vaginal: ↑Clostridium butyricum, C. disporicum, Alloscardovia omnicolens, Veillonella montpellierensis; ↓Lactobacillus jensenii, L. reuteri, L. iners; ↑alpha diversity in EM/AM-CPP vs other groups	Diagnostic biomarker signature for CPP; pain not formally scored
Facciotti *et al.* (2025)	Systematic scoping review	36 studies	Endometriosis	Multi-site (vaginal, cervical, gut, peritoneal, uterine) inconsistent genus-level signals (↑Streptococcus in cervical fluid, ↑Pseudomonas in peritoneal fluid and ↓Lachnospira in stool/anal fluid; low reproducibility across cohorts	No consistent pain outcome data; methodological heterogeneity highlighted
Muraoka *et al.* (2023)	Clinical + preclinical	Human cohort (n=155; FISH/IHC subset n=84); syngeneic mouse model	Endometriosis	Endometrial: *Fusobacterium nucleatum* enriched in ∼64% endometriosis vs ∼7% controls; antibiotic treatment reduced lesion burden in mice	No direct pain outcome; non-reproduced in independent cohorts; illustrative of replication challenges
Wessels *et al.* (2021)	Observational	Clinical cohort (n=21)	Endometriosis	Endometrial: ↑Shannon diversity in endometriosis; discriminant taxa included ↑Actinobacteria/Streptococcaceae/Tepidimonas in endometriosis and ↑Burkholderiaceae/Ralstonia in controls.	Pain not directly scored; findings suggest perturbation of the endometrial microbiota in endometriosis
T. Chen *et al.* (2021)	Preclinical (rat model)	Rat vaginal dysbiosis model (n=24)	Vaginal dysbiosis model	Vaginal dysbiosis: ↓Lactobacillus, ↑Enterobacter/Enterococcus; VMT or Lactobacillus probiotic mix restored Lactobacillus dominance and reduced vaginal IL-1β/TNF-α	Mucosal inflammation reduced after VMT; pain behaviour not assessed

Summary of studies examining vaginal and endometrial microbiota in relation to CPP conditions. BV, bacterial vaginosis; endo, endometriosis; VMT, vaginal microbiota transplantation.

Abbreviations: AUB, abnormal uterine bleeding; BV, bacterial vaginosis; CA125, cancer antigen 125; CPP, chronic pelvic pain; CXCL1/GRO, C-X-C motif chemokine ligand 1/growth-regulated oncogene; EM/AM, endometriosis/adenomyosis; FRT, female reproductive tract; IBS, irritable bowel syndrome; IL, interleukin; MDC, macrophage-derived chemokine; TNF, tumour necrosis factor; VEGF, vascular endothelial growth factor; VMT, vaginal microbiota transplantation.

#### Urinary ecosystem

The urinary tract harbours a specific resident microbiota (Whiteside *et al.*, 2015; Palumbo *et al.*, 2025). Spatial profiling demonstrates that urine and urothelium contain related but distinct microbial communities, with *Lactobacillus* spp. dominating urine and *Staphylococcus* spp. more abundant in the urothelium (Wolfe *et al.*, 2023). Studies investigating BPS/IC show heterogeneous results, with some reporting differences in alpha diversity, *Lactobacillus* spp. relative abundance or urinary metabolites (Walton and Nickel, 2021; Fu *et al.*, 2024). Etiocholanolone sulphate, an excretory product of testosterone metabolism, was identified in the urinary metabolome as a highly discriminatory metabolite for BPS/IC, separating patients from controls with >90% accuracy. Importantly, its levels correlated with pelvic pain intensity and these alterations remained stable over 3-6 months, suggesting a persistent biochemical endophenotype in severe BPS/IC (Parker *et al.*, 2016).

Subtype-specific differences are increasingly apparent, with Hunner-type IC, defined by the presence of characteristic inflammatory Hunner lesions on cystoscopy, being associated with increased levels of *Pseudomonas* and *Gardnerella*, whereas non-Hunner IC shows a higher relative abundance of *Lactobacillus* spp. and *Enterococcus* spp. (Zhu *et al.*, 2025). Integrated urine microbiome–metabolome profiling highlights a distinct BPS/IC urinary microenvironment, with shifts in community structure (notably altered β-diversity) and dozens of differentially abundant genera and metabolites (Zheng *et al.*, 2023). These paired signatures include depletion of potentially protective taxa, like *Lactobacillus* spp., alongside changes in inflammation-relevant compounds, most notably reduced theophylline, supporting microbe–metabolite interactions as plausible contributors to symptom-associated inflammatory pathways (Zheng *et al.*, 2023). Interventional evidence suggests microbial plasticity: dextrose prolotherapy appears to shift urinary profiles towards more favourable compositions and improve symptoms (Chen *et al.*, 2025).

Key studies examining the urinary microbiota and metabolome in relation to CPP are summarized in Table 2.

**Table 2.** Urinary microbiome and metabolome studies.

Reference	Study design	N/Model	CPP condition	Key microbial findings	Pain/clinical outcome
Zhu *et al.* (2025)	Observational multi-omics	Clinical cohort (n=30)	IC/BPS (Hunner vs non-Hunner)	Urine/urothelium: Hunner-IC ↑*Pseudomonas*, *Gardnerella*; non-Hunner ↑*Lactobacillus*, *Enterococcus*	Subtype-specific microbial signatures; Hunner-IC associated with greater inflammatory burden; pain not directly correlated with microbiota
Zheng *et al.* (2023)	Observational (microbiome + metabolome)	Clinical cohort (n=60)	IC/BPS	Urine: altered β-diversity, Lactobacillus, ↑Sphingomonas; ↓theophylline; dozens of differentially abundant genera/metabolites	Microbe–metabolite interactions plausibly contribute to symptom-associated inflammation
Parker *et al.* (2016)	Longitudinal observational	Clinical cohort (n=80)	IC/BPS	Urinary metabolome: etiocholanolone sulphate highly discriminatory (>90% accuracy)	Metabolite levels correlated with pelvic pain intensity and symptom burden; stable over 3–6 months
Walton and Nickel (2021)	Critical review	6 studies	IC/BPS	Urinary microbiome: heterogeneous results on alpha diversity and *Lactobacillus* abundance	Current evidence insufficient to establish causal role; methodological limitations highlighted
Wolfe *et al.* (2023)	Spatial profiling study	Clinical cohort (n=40)	IC/BPS	Bladder biopsy: ↑Staphylococcus (most abundant), Lactobacillus second; no significant difference between IC/BPS and controls	Bacteria detected in bladder urothelium but no IC/BPS-specific dysbiotic pattern; no pain correlation
L.-K. Chen *et al.* (2025)	Interventional (pilot)	Clinical cohort (n=77)	IC/BPS	Urinary: ↑Proteobacteria, Firmicutes, Bacteroidota in IC/BPS; dextrose prolotherapy: ↓Ureaplasma, ↑Lactococcus, ↑L. lactis	Symptom improvement observed post-intervention; small sample, preliminary

Summary of studies examining the urinary microbiota and metabolome in relation to IC/BPS and CPP.

Abbreviations: BPS, bladder pain syndrome; CPP, chronic pelvic pain; HIC, Hunner interstitial cystitis; IC, interstitial cystitis; IC/BPS, interstitial cystitis/bladder pain syndrome; NHIC, non-Hunner interstitial cystitis; β-diversity, between-sample microbial diversity.

#### Gut ecosystem

The gut contains various receptors and ion channels involved in nociceptive signalling, including transient receptor potential (TRP) channels, serotonergic, and cannabinoid pathways. Gut dysbiosis is one of the most consistent findings in pelvic pain disorders and gut microbiota can activate these receptors directly or indirectly (Rea *et al.*, 2019). These pathways involve Toll-like receptors, TRP channels, opioid receptors, and serotonergic signalling (Guo *et al.*, 2019). Women with endometriosis, IBS, or mixed-phenotype CPP frequently exhibit reduced microbial diversity and enrichment of Gram-negative anaerobes, particularly members of the Bacteroidetes phylum, such as members of *Prevotella*, *Bacteroides*, *Alistipes*, and *Parabacteroides* genera (Li *et al.*, 2022; Hearn-Yeates *et al.*, 2024; Ren *et al.*, 2025).

Environmental factors strongly modulate gut dysbiosis risk. Post-infectious IBS develops in ∼21% of individuals following *Campylobacter* infection, particularly in women, younger individuals and those with severe initial symptoms (Berumen *et al.*, 2021). Antibiotic use further contributes to dysbiosis, nearly doubling the odds of IBS development (Krogsgaard *et al.*, 2018). Functionally, gut dysbiosis is accompanied by metabolic alterations, including elevated primary and conjugated bile acids that correlate with pain severity and differ across IBS subtypes (Dior *et al.*, 2016), as well as reduced short-chain fatty acids (SCFAs), particularly butyrate. SCFA depletion contributes to barrier dysfunction, increased cytokine production and nociceptive sensitization (Chadchan *et al.*, 2021; Li *et al.*, 2022; Ustianowska *et al.*, 2022; Hearn-Yeates *et al.*, 2024; Ren *et al.*, 2025). In murine models of endometriosis, antibiotic-mediated depletion of the gut microbiota significantly reduces lesion number, volume, angiogenesis, and macrophage infiltration, whereas restoration via faecal microbiota transfer from diseased donors rescues lesion growth (Chadchan *et al.*, 2019, 2023). Furthermore, transplantation experiments using germ-free donor tissue show that uterine-associated microbiota is dispensable for lesion development, implicating intestinal microbial communities as dominant regulators of pelvic inflammatory tone (Yuan *et al.*, 2018; Chadchan *et al.*, 2023).

Mechanistic studies in pain biology support a role for the gut microbiota in nociceptive modulation. Depletion of the gut microbiota in germ-free or antibiotic-treated mice reduces mechanical hypersensitivity in neuropathic pain models. Restoration of microbiota normalizes microglial maturation and re-establishes pain behaviour, in parallel with increased pro-inflammatory cytokine production and Toll-like receptor-dependent NF-κB signalling in spinal microglia (Lin *et al.*, 2020).

Key studies examining gut microbiota in relation to CPP and related conditions are summarized in Table 3.

**Table 3.** Gut microbiome studies in CPP-related conditions.

Reference	Study design	N/Model	CPP condition	Key microbial findings	Pain/clinical outcome
Hearn-Yeates *et al.* (2024)	Narrative review	Multiple studies	Endometriosis-associated CPP	No consensus taxa; microbiota–gut–brain axis proposed as a potential pathway linking dysbiosis with pain, GI symptoms and mood disorders in endometriosis	No formal pain correlation concluded; methodological heterogeneity highlighted
Pronina *et al*., 2025	Prospective before–after pilot cohort	Clinical cohort (n=17)	Endometriosis	Gut: ↓Bacillota/Bacteroidota ratio; ↓Staphylococcus spp.; ↑Lactobacillus spp. and Collinsella aerofaciens after ≥6 months of dienogest; non-significant ↑alpha diversity	No formal pain–microbiome correlation; no vaginal microbiome analysis.
Ren *et al.* (2025)	Mechanistic synthesis (review)	Multiple studies	CPP, endometriosis, IBS	Gut: metabolites, immune pathways and direct nociceptor activation implicated; probiotics/FMT/diet discussed as translational strategies	Convergent mechanistic evidence for gut-driven neuroimmune sensitization in CPP; no direct pain score data
Chadchan *et al.* (2021)	Preclinical (murine model)	Mouse model	Endometriosis	Gut: endometriosis-associated dysbiosis with reduced fecal n-butyrate; microbiota depletion reduced lesion growth, FMT from endometriosis mice restored lesions, and n-butyrate supplementation reduced lesion burden	SCFAs, especially n-butyrate, protect against lesion progression; no direct pain outcome
Chadchan *et al.* (2023)	Preclinical (murine model)	Mouse model	Endometriosis	Gut: germ-free donor tissue experiments—uterine microbiota dispensable for lesion development	Intestinal microbiota identified as dominant regulator of pelvic inflammatory tone; no direct pain outcome
Yuan *et al.* (2018)	Preclinical (murine model)	Mouse model	Endometriosis	Gut: ↑Firmicutes/Bacteroidetes ratio, ↑Bifidobacterium at 42 days post-induction; similar alpha diversity between groups	Dysbiosis tracks disease progression; no direct pain outcome
Dior *et al.* (2016)	Observational	Clinical cohort (n=46)	IBS subtypes	Gut: ↑E. coli in IBS-D; ↑Bacteroides, ↑Bifidobacterium in IBS-C; ↑primary and amino-conjugated bile acids in both subtypes; ↓bile acid deconjugation activity	Bile acid alterations correlated with abdominal pain severity across IBS subtypes
Lin *et al.* (2020)	Narrative review	Multiple studies	Neuropathic pain/gut–brain axis	Gut: microbiota regulates NP via immune, neural, endocrine and metabolic pathways	Gut microbiota implicated in central pain sensitisation; therapeutic strategies include probiotics and dietary interventions
Rahman-Enyart *et al.* (2021)	Preclinical (murine model)	Mouse model	Pelvic pain/LPS model	Gut: impaired LPS detoxification → exaggerated pelvic pain; reversible by microbiota transfer	Pelvic pain behaviour assessed; microbiota–LPS axis drives nociception
Cardaillac *et al.* (2025)	Observational multi-omics	Clinical cohort (n=28)	Sensitized CPP (S-CPP)	Gut + vaginal + urinary: ↓*Lactobacillus* across compartments; *Peptostreptococcales-Tissierellales* and *Christensenellaceae_R-7* modules correlated with pain + anxiety	Multi-compartment dysbiosis associated with pain intensity, anxiety, and GI symptoms
Berumen *et al.* (2021)	Prospective cohort	Clinical cohort (n=1667)	Post-infectious IBS	Gut: Campylobacter infection → IBS in ∼21%; dysbiosis as post-infectious sequela	Abdominal pain and bowel symptoms; female sex and symptom severity as risk factors
Guo *et al.* (2019)	Mechanistic review (preclinical evidence synthesis)	Multiple preclinical studies	Visceral/neuropathic pain	Gut: molecular pathways linking microbiota to nociception via TLRs, TRPV channels, opioid receptors, serotonergic pathways	Foundational mechanistic framework for microbiota–nociceptive signalling; no direct pain score data

Summary of clinical and preclinical studies examining the gut microbiota in relation to endometriosis, IBS, and CPP.

Abbreviations: CPP, chronic pelvic pain; FMT, faecal microbiota transplantation; GI, gastrointestinal; IBS, irritable bowel syndrome; IBS-C, constipation-predominant irritable bowel syndrome; IBS-D, diarrhoea-predominant irritable bowel syndrome; LPS, lipopolysaccharide; NP, neuropathic pain; SCFA, short-chain fatty acid; S-CPP, sensitized chronic pelvic pain; α-diversity, within-sample microbial diversity.

### Barrier dysfunction and mucosal immune activation

Dysbiosis across pelvic compartments destabilizes epithelial barriers and drives pro-inflammatory immune reprogramming. Lipopolysaccharide (LPS) and other microbial components activate Toll-like receptor 4 (TLR4), triggering nuclear factor kappa B (NF-κB), and cyclooxygenase-2 (COX-2) signalling, increasing IL-6, IL-1β, and TNF-α levels while weakening tight-junction integrity (Li *et al.*, 2022; Ustianowska *et al.*, 2022; Guo and Zhang, 2024; Ren *et al.*, 2025). In a rat model of vaginal dysbiosis, loss of *Lactobacillus* spp. and anaerobes overgrowth triggered mucosal inflammation with increased IL-1β and TNF-α expression, which could be reversed by vaginal microbiota transplantation or selected probiotics (Chen *et al.*, 2021).

Fungal dysbiosis may further modulate symptom severity. Recurrent vulvovaginal candidiasis is strongly associated with vulvodynia, consistent with β-glucan signalling (De Seta *et al.*, 2022) through Dectin-1 and Toll-like receptor 2 (TLR2) pathways (Ren *et al.*, 2025). These mechanisms may impair *Lactobacillus* persistence and epithelial homeostasis (De Seta *et al.*, 2022).

Endometriosis itself is characterized by broad immune dysregulation involving macrophages, dendritic cells, mast cells, regulatory T cells, and natural killer (NK) cells, which synergize with microbial cues to maintain chronic inflammation (Garmendia *et al.*, 2025). In animal models, microbiota depletion reduces mechanical and visceral hypersensitivity, whereas recolonization with a complex microbiota restores pain behaviour alongside microglial maturation and pro-inflammatory cytokine production in the spinal cord (Lin *et al.*, 2020). Consistent with this, mice with impaired detoxification of LPS develop exaggerated pelvic pain that is reversible following microbiota transfer (Rahman-Enyart *et al.*, 2021). Together, these findings suggest that gut microbiota-derived immune signals can amplify peripheral pelvic nociception while reinforcing central sensitization, thereby contributing to pain persistence independently of lesion burden.

### Endocrine modulation and microbial metabolism

The estrobolome, defined as the repertoire of gut microbial genes encoding β-glucuronidase (GUS) activity, regulates systemic estrogen recirculation by deconjugating hepatic estrogen glucuronides in the intestine, allowing their reabsorption into the enterohepatic circulation and subsequent distribution to peripheral target tissues. This is particularly relevant in estrogen-sensitive disorders such as endometriosis and adenomyosis, where estrogen-dependent signalling sustains inflammation and nociception (Hu *et al.*, 2023). Dominant GUS-producing taxa include *Escherichia*, *Bacteroides*, and *Clostridium*, along with other members of the Firmicutes lineage (Ervin *et al.*, 2019; Hu *et al.*, 2023). Subsequent experimental studies confirmed that enhanced microbial GUS activity increases the pool of deconjugated estrogens and promotes pro-inflammatory cytokine release, including IL-6 and IL-1β, thereby linking estrogen recycling to immune activation (Baker *et al.*, 2017; Alghetaa *et al.*, 2023; Pai *et al.*, 2023). In women with endometriosis, gut microbiome studies suggest an increased abundance of GUS-producing bacteria, particularly *Escherichia* and *Bacteroides* members (Jiang *et al.*, 2021). However, direct evidence linking dysbiosis to circulating estrogen levels remains limited. Whether aromatase inhibitors or progestins modulate microbiome-driven estrogen reactivation remains largely unknown. In a small prospective before–after cohort of women with endometriosis, dienogest therapy was associated with changes in gut microbial composition, including a reduced Bacillota/Bacteroidota ratio, decreased Staphylococcus spp. and increased Lactobacillus spp. and Collinsella aerofaciens, suggesting that progestin therapy may influence intestinal microbial communities. However, this study did not assess vaginal microbiota, microbial estrogen reactivation or direct pain–microbiome correlations (Pronina *et al.*, 2025). This represents an important mechanistic gap warranting prospective investigation integrating microbial, hormonal, and pain phenotyping.

Exogenous hormonal contraception modestly modulates the vaginal microbiome in a method-dependent manner: estrogen-containing rings promote *Lactobacillus* spp. dominance (Crucitti *et al.*, 2018), whereas long-term use of depot medroxyprogesterone acetate may reduce it (Mitchell *et al.*, 2014), with little net effect observed across injectable and intrauterine methods (Jacobson *et al.*, 2014; Achilles *et al.*, 2018).

Tryptophan metabolism constitutes another bidirectional pathway linking dysbiosis to immune activation and nociception. Indole derivatives such as indole-3-acetate, indole-3-lactate, and indolepropionic acid signal through the aryl hydrocarbon receptor (AhR) to enhance IL-22-mediated epithelial barrier integrity (Escorcia Mora *et al.*, 2025). In inflammatory states, diversion towards the kynurenine pathway is associated with immune activation and N-methyl-D-aspartate receptor-dependent pain sensitization (Ren *et al.*, 2025). Yet this pathway is not exclusively pro-inflammatory, as certain kynurenines can activate G protein-coupled receptor 35 and exert antinociceptive and immunomodulatory effects (Li *et al.*, 2022). Overall, gut microbiota-dependent tryptophan metabolism—particularly the balance between the serotonin and kynurenine pathways—may exert context-dependent effects on gut–brain signalling and neuroimmune regulation (Qu *et al.*, 2024). In addition, gut microbiota can modulate neurotransmitters including dopamine, glutamate, gamma-aminobutyric acid (GABA), and neurosteroids like allopregnanolone that may be linked to pain (McCurry *et al.*, 2024).

### Microbiome-driven neuroimmune circuits and central sensitization in CPP

Microbiome-derived signals interact with neural pathways via the gut–brain axis to drive visceral and pelvic pain. Gut dysbiosis enhances microglial activation and increases central IL-6 and IL-1β levels in experimental mouse models (Griffiths *et al.*, 2024; Ren *et al.*, 2025). Advanced tracing and chemogenetic tools used to map and selectively activate enteric neurons in mice demonstrate that perturbation of gut–neural circuits reshapes mucosal immunity, microbial metabolite profiles, and host–microbiome signalling, providing causal evidence for bidirectional neuroimmune communication (Griffiths *et al.*, 2024). Human neuroimaging studies in IBS consistently demonstrate hyperactivation of key pain-processing regions, including the anterior cingulate cortex, insula, prefrontal cortex, and thalamus, alongside impaired descending pain modulation (Hubbard *et al.*, 2015; Wang *et al.*, 2017). Experimental suppression of cingulate cortical activity in murine models attenuates visceral hypersensitivity and anxiety-like behaviours (Brenner *et al.*, 2021).

Neuromodulation strategies may alleviate pelvic and visceral pain partly through microbiota-dependent mechanisms. In a single-blind randomized controlled trial (RCT) in constipation-predominant irritable bowel syndrome (IBS-C) (n = 40), transcutaneous auricular vagus nerve stimulation (taVNS) significantly improved abdominal pain, bowel habits, and psychological scores, while increasing vagal tone (Liu *et al.*, 2024). Consistently, psychobiotics such as *Bifidobacterium breve* and *Bifidobacterium longum* have been shown to modulate central stress and emotional circuits via modulation of the hypothalamic–pituitary–adrenal (HPA) axis (Dinan and Cryan, 2017; Ķimse *et al.*, 2024).

However, microbiota-mediated analgesic effects are strain- and context-dependent. While *L. reuteri* restores peripheral opioid receptor expression and reduces visceral hypersensitivity in distension models (Hegde *et al.*, 2020), other strains fail to produce analgesia in neuropathic or inflammatory pain, with no effect on microglial activation (Huang *et al.*, 2019).

### Microbial metabolites as integrators of epithelial, immune, and neural pathways

Microbial metabolites link microbial community structure to epithelial integrity, immune regulation, and neural sensitization in CPP. SCFAs, mainly butyrate, acetate, and propionate, promote regulatory T-cell differentiation, suppress pro-inflammatory cytokines such as IL-6, TNF-α, and IL-17A, and attenuate microglial activation by inhibiting TLR4–MyD88–NF–κB signalling, thereby limiting neuroinflammation along gut–brain pain circuits in experimental models (Huuskonen *et al.*, 2004; Du *et al.*, 2024; Facchin *et al.*, 2024; Zhao *et al.*, 2025). Bile acids modulate visceral sensitivity and are altered in IBS with increased primary and conjugated bile acids correlating with pain severity (Dior *et al.*, 2016).

Tryptophan-derived metabolites influence both immune and neural processes. Multi-omics analyses identified several indole derivatives, including 4-hydroxyindole, as reduced in endometriosis and functional experiments demonstrated that 4HI exerts anti-inflammatory and anti-nociceptive effects while suppressing lesion initiation and progression in murine and human xenograft models (Talwar *et al.*, 2025). In a recent single cohort metabolomic study, Proteobacteria-associated alterations in glycerophospholipids, particularly phosphatidylcholine PC(40:8), achieved near-perfect discrimination between women with endometriosis/adenomyosis and controls (Li *et al.*, 2025), supporting the diagnostic potential of integrated microbial–metabolite signatures.

### Mechanistic integration and causal evidence for microbiota-driven pelvic pain

Together, these findings suggest that dysbiosis may not merely correlate with pelvic pain, but may contribute to shaping immune, metabolic, and neural pathways. Experimental manipulations in preclinical models provide mechanistic evidence for bidirectional interactions between microbiome and chronic pain, although direct causal evidence in humans remains limited. Beyond endometriosis, systems-biology analyses in women with sensitized CPP (S-CPP), including mixed-phenotype CPP, demonstrate a consistent depletion of *Lactobacillus* genera and ASVs across the gut, vaginal, and urinary microbiomes. In parallel, taxonomic modules dominated by *Peptostreptococcales-Tissierellales* and *Christensenellaceae_R-7* correlate with pain intensity, anxiety, and gastrointestinal symptoms, supporting coordinated microbial networks as potential drivers of pelvic sensitization (Cardaillac *et al.*, 2025). Multi-omics frameworks integrating taxonomic, functional, and metabolite data can identify disease-associated modules and link them to host immune-metabolic pathways, supporting precision-medicine applications (Muller *et al.*, 2024; Yang *et al.*, 2025).

Among pelvic pain disorders, endometriosis provides the most experimentally tractable model to investigate microbiota–pain interactions, with well-established systems allowing manipulation of both microbial communities and nociceptive outcomes. Induction of endometriosis in mice alters gut microbiota composition, including shifts in the Firmicutes/Bacteroidetes ratio and increased *Bifidobacterium* abundance at later disease stages (Yuan *et al.*, 2018). Integrated metabolomic analyses further reveal reduced microbial diversity and coordinated alterations in secondary bile acid biosynthesis and α-linolenic acid metabolism, suggesting that dysbiosis reshapes bioactive lipid and bile acid pools (Ni *et al.*, 2020). In a murine model, broad-spectrum antibiotics reduce lesion burden and inflammatory status, whereas faecal microbiota transfer from mice with endometriosis restores disease activity, supporting a causal contribution of the gut microbiota (Chadchan *et al.*, 2019). At the genital level, preliminary evidence from a murine preprint, not yet peer-reviewed, suggests that endometriosis-associated vaginal dysbiosis may be sufficient to induce pain (Pratt *et al.*, 2025). In the same model, intravaginal antibiotic treatment and vaginal microbiome transplantation (VMT) from healthy donors transiently alleviate mechanical allodynia and ongoing pain in endometriosis mice, while VMT from endometriosis donors induces allodynia and spontaneous nociceptive behaviours in otherwise healthy recipients (Pratt *et al.*, 2025). These findings suggest a potential role for vaginal microbiota-driven pain transmission, but should be interpreted with appropriate caution until confirmed in peer-reviewed and translational studies.

### Therapeutic approaches targeting microbiome-host functional axes

Preclinical models consistently demonstrate that microbiome-targeted interventions can reduce lesion burden, visceral hypersensitivity and inflammatory tone. However, clinical evidence remains limited: only a small number of RCT have been conducted, most in IBS rather than CPP specifically, and effect sizes are modest with substantial heterogeneity across studies. No intervention has yet demonstrated sufficient efficacy and safety to be recommended as standard-of-care in CPP management. A structured summary of these interventions by level of evidence is provided in Table 4.

**Table 4.** Therapeutic interventions targeting the microbiome in CPP and related conditions.

Reference	Study design	N/Population	Intervention	Compartment targeted	Main outcome
Khodaverdi *et al.* (2019)	RCT (pilot)	n = 37, endometriosis stage III–IV	Oral multi-strain *Lactobacillus* spp. supplementation	Gut/systemic	↓dysmenorrhea and overall pain vs placebo; limited by small n, short FU
Goodoory *et al.* (2023)	Meta-analysis (82 RCTs)	n = 10 332, IBS	Probiotics (various strains)	Gut	Modest strain-specific benefit on global symptoms/pain; low-to-very-low certainty; high heterogeneity
Martoni *et al.* (2020)	RCT (multicentre)	n = 330, IBS	*L. acidophilus* DDS-1 + *B. lactis* UABla-12	Gut	Clinically meaningful ↓abdominal pain; excellent tolerability
Ait-Belgnaoui *et al.* (2018)	Preclinical (murine model)	Mouse model	Probiotics (*B. longum* + *L. helveticus*)	Gut/HPA axis	↓stress-induced visceral hypersensitivity via HPA modulation
Itoh *et al.* (2011)	Preclinical (murine model)	Mouse model	*Lactobacillus* spp. probiotics	Gut	↓ectopic lesion growth via IL-12-dependent NK-cell activation; no direct pain outcome
Bertin *et al.* (2024)	Narrative review	IBS patients	Low-FODMAP diet	Gut	↓abdominal pain and bloating; alters SCFA profiles; modest evidence quality
Nirgianakis *et al.* (2022)	Systematic review	Endometriosis	Multiple dietary interventions	Gut/systemic	Most studies report positive effect on endo symptoms; moderate-to-high risk of bias
Rodríguez-Daza *et al.* (2021)	Narrative review	Multiple studies	Polyphenol-rich diet (berries, grapes)	Gut	Mechanistic evidence for polyphenol-mediated microbiota modulation; no direct pain outcome
Aroniadis *et al.* (2019)	DB RCT crossover	n = 48, IBS-D	FMT capsules	Gut	No significant difference in IBS-SSS at 12 weeks (221 vs 236, p=0.65); strong placebo response
Pratt *et al.* (2025) **—VMT arm (preprint)**	Preclinical (murine model)	Mouse model (non-surgical endo)	VMT	Vaginal	VMT from healthy donors ↓allodynia; VMT from endometriosis donors transfers pain phenotype to naïve recipients. No clinical RCT
Martinelli *et al.* (2023)	Narrative review	Multiple gynecological conditions	VMT	Vaginal	Summarises FMT/VMT evidence for gynecological disorders; clinical VMT; no CPP-specific or pain outcome data
Liu *et al.* (2024)	Single-blind RCT	n = 40, IBS-C	Transcutaneous auricular vagus nerve stimulation (taVNS)	Gut–brain axis	↓abdominal pain; ↑vagal tone; ↑*Bifidobacterium*, ↑SCFAs, ↓tryptophan metabolites
Ervin *et al.* (2019), Hu *et al.* (2023)	Preclinical (experimental + review)	Animal models + review	Selective β-glucuronidase inhibitors	Gut/estrobolome	↓estrogen reactivation; modulates enterohepatic cycling; potential relevance to endo/CPP; no direct pain outcome; no clinical trials yet
Chadchan *et al.* (2021)	Preclinical (murine model)	Mouse model (surgical + injection-based endo)	n-butyrate / SCFAs	Gut	↓fecal n-butyrate in endo mice; n-butyrate ↓lesion growth via HDAC inhibition and GPR signalling; no direct pain outcome
Chadchan *et al.* (2019)	Preclinical (murine model)	Mouse model (surgically-induced endo)	Broad-spectrum oral antibiotics / metronidazole	Gut	∼5-fold ↓lesion size; metronidazole alone sufficient; FMT from endo mice restored lesion growth; ↓peritoneal inflammation; no direct pain outcome
Muraoka *et al.* (2023)	Preclinical (murine model) + clinical cohort	n=155 + murine model	Targeted antibiotics (*F. nucleatum*-directed)	Endometrial/gut	↓lesion burden in antibiotic-treated mice. Human cohort showed increased prevalence of *F. nucleatum* in endometriosis patients; finding not independently reproduced. No clinical RCT; off-label use not supported.
Pratt *et al.* (2025) **—antibiotic arm (preprint)**	Preclinical (murine model)	Mouse model (non-surgical endo)	Intravaginal antibiotics	Vaginal	Intravaginal antibiotics ↓abdominal mechanical allodynia and ongoing pain transiently. No clinical RCT; off-label use not supported.

Summary of studies evaluating microbiome-targeted interventions, including probiotics, diet, faecal or vaginal microbiota transplantation, antibiotics, SCFAs and microbiota-related neuromodulation.

Abbreviations: BV, bacterial vaginosis; CPP, chronic pelvic pain; DB, double-blind; endo, endometriosis; FMT, faecal microbiota transplantation; FODMAP, fermentable oligosaccharides, disaccharides, monosaccharides and polyols; FU, follow-up; GPR, G-protein-coupled receptor; HDAC, histone deacetylase; HPA, hypothalamic–pituitary–adrenal; IBS, irritable bowel syndrome; IBS-C, constipation-predominant irritable bowel syndrome; IBS-D, diarrhoea-predominant irritable bowel syndrome; NK, natural killer; RCT, randomised controlled trial; SCFA, short-chain fatty acid; taVNS, transcutaneous auricular vagus nerve stimulation; VMT, vaginal microbiota transplantation.

Probiotics demonstrate anti-inflammatory and analgesic effects in preclinical endometriosis and IBS models, including reduced stress-induced visceral hypersensitivity via HPA-axis modulation and suppression of ectopic lesion growth via IL-12-dependent NK-cell activation (Itoh *et al.*, 2011; Ait-Belgnaoui *et al.*, 2018; Zhou *et al.*, 2020). In a small pilot randomized trial in women with stage III–IV endometriosis (n = 37), oral multi-strain *Lactobacillus* spp. supplementation was associated with greater short-term reductions in dysmenorrhea and overall pain scores, although sample size and follow-up were limited (Khodaverdi *et al.*, 2019). A recent meta-analysis of 82 RCTs in IBS (n = 10 332) reported modest, strain-specific benefits of selected *Bifidobacterium* and *Lactobacillus* formulations on global symptoms and abdominal pain, but with low certainty of evidence and substantial heterogeneity across strains and combinations (Goodoory *et al.*, 2023). Among individual strains, excellent tolerability and clinically meaningful reductions in abdominal pain in IBS have been demonstrated for *Lactobacillus acidophilus* DDS-1 and *Bifidobacterium lactis* UABla-12 in a large multicentre RCT (n = 330) (Martoni *et al.*, 2020). Whether probiotic or prebiotic interventions can durably reverse established dysbiosis remains unclear, as colonization resistance may limit sustained engraftment and long-term microbiome recovery is rarely demonstrated (Suez *et al.*, 2018; Zmora *et al.*, 2018).

Dietary interventions modulate the gut microbiome function through converging mechanisms. Low fermentable oligosaccharides, disaccharides, monosaccharides, and polyols (FODMAP) diets (dietary approaches that reduce intake of specific fermentable carbohydrates) reduce abdominal pain and bloating in IBS while altering fermentation patterns and SCFA profiles, although evidence remains modest (Bertin *et al.*, 2024). Fibre- and polyphenol-rich interventions, ranging from targeted additions (nuts, legumes, cocoa, and berries) to whole-diet patterns, consistently enrich SCFA-producing genera such as *Faecalibacterium*, *Roseburia*, *Eubacterium*, and *Blautia*, and shift circulating and faecal metabolites towards anti-inflammatory profiles (Meiners *et al.*, 2025;  Rodríguez-Daza *et al.*, 2021). Emerging data in endometriosis suggest that diet-induced shifts in the gut microbiota, particularly with Mediterranean/anti-inflammatory and low-FODMAP patterns, may contribute to pain relief via effects on inflammation, mast-cell activation, and estrogen signalling, although mechanistic evidence remains largely indirect and based on small or uncontrolled studies (Moore *et al.*, 2017; Nirgianakis *et al.*, 2022; Abulughod *et al.*, 2024; Türkoğlu *et al.*, 2025). Postbiotic SCFAs act as signalling molecules with direct G protein-coupled receptor and histone deacetylase-mediated anti-inflammatory and neuromodulatory effects (Du *et al.*, 2024; Zhao *et al.*, 2025). Other strategies target the hormonal–microbiome interface, notably selective inhibitors of gut microbial β-glucuronidases, which modulate enterohepatic estrogen cycling (Ervin *et al.*, 2019; Hu *et al.*, 2023). Psychobiotics, encompassing specific probiotics and prebiotics targeting the gut–brain axis, modulate HPA-axis activity, systemic inflammation, and neurotransmitter pathways, with preclinical models and small clinical trials suggesting improvements in mood and stress-related symptoms and a possible reduction in visceral hypersensitivity, although clinical evidence remains heterogeneous (Dinan and Cryan, 2017; Ķimse *et al.*, 2024).

Microbiota transplantation approaches represent ecosystem-level interventions with variable translational signals across indications. Preclinical data suggest that microbial communities can drive pain independently of lesion burden. In murine endometriosis models, vaginal dysbiosis can induce pain: while antibiotics and VMT from healthy donors alleviate allodynia, VMT from diseased donors transfers pain phenotypes (Pratt *et al.*, 2025). By contrast, clinical translation in IBS has been disappointing. In a rigorously designed double-blind, randomized, placebo-controlled crossover trial of faecal microbiota transplantation capsules in diarrhoea-predominant IBS (N = 48), no significant difference in symptom severity was observed between faecal microbiota transplantation and placebo at 12 weeks (Aroniadis *et al.*, 2019). These findings highlight strong placebo responses, methodological heterogeneity, and the need for improved patient stratification and mechanism-based endpoints. By contrast, VMT shows promise in recurrent bacterial vaginosis, with early data supporting feasibility and microbiome restoration (Chen *et al.*, 2021; Martinelli *et al.*, 2023). Next-generation strategies are moving towards defined synthetic microbial consortia and engineered bacteria designed to restore specific metabolic pathways, or to deliver inducible anti-inflammatory effectors, sometimes combined with phage-based approaches (Lynch *et al.*, 2025; Mkilima, 2025). These live biotherapeutic approaches will nevertheless require rigorous manufacturing, strain-level characterization, antimicrobial-resistance surveillance, and long-term pharmacovigilance frameworks to ensure safety and regulatory acceptability (Lynch *et al.*, 2025).

Antibiotics occupy a paradoxical role in the microbiome-CPP landscape: preclinical models demonstrate that broad-spectrum gut microbiota depletion reduces endometriosis lesion burden and visceral hypersensitivity, and intravaginal antibiotics transiently alleviate pain in vaginal dysbiosis models (Chadchan *et al.*, 2019; Pratt *et al.*, 2025). However, clinical use carries significant risks—disruption of commensal communities, worsening dysbiosis, post-infectious IBS, and antimicrobial resistance—and no adequately powered trials support their use for CPP-associated dysbiosis. Antibiotics should therefore be reserved for documented infections rather than empirical dysbiosis management.

## Discussion, limitations, and future directions

This review highlights substantial heterogeneity and variable quality in the existing evidence base. Most human studies are small, cross-sectional, and rely on variable case definitions, sampling strategies, and sequencing methodologies, often with incomplete metadata on key confounders such as antibiotic exposure, hormonal therapies, diet, menstrual cycle phase, and menopausal status. Low-biomass pelvic sites, including the endometrium and bladder, are particularly vulnerable to contamination and current research remains largely bacteria-centric, with sparse and preliminary data on the virome and mycobiome. Collectively, these factors constrain causal inference and limit direct comparability across studies, in addition to generally low participant numbers. Correlation with pain symptoms remains limited, as most studies investigate the microbiome primarily as a disease signature rather than in relation to symptomatology. This pattern is frequently observed in endometriosis research. It is also worth noting that dysbiosis across gut, vaginal, and endometrial compartments is also observed in asymptomatic women, suggesting it is insufficient alone to drive CPP. Additional host factors—immunological, neurological, or genetic—likely shape clinical expression. Moreover, most human evidence remains associative, with causal insights largely derived from preclinical models.

Future research should prioritize longitudinal and interventional designs that sample multiple pelvic compartments, gut, vagina, bladder, and endometrium, within the same individuals and integrate microbiome data with immune, endocrine, neural, and psychosocial phenotyping. Harmonized protocols and reporting standards, ideally aligned with STORMS (Strengthening The Organization and Reporting of Microbiome Studies) recommendations, together with multi-omics approaches, will be essential to identify robust microbial and metabolite signatures that cut across CPP syndromes. Mechanism-based trials of dietary, probiotic/psychobiotic, postbiotic, and microbial transfer interventions should embed predefined microbial and host biomarkers alongside pain outcomes, enabling patient stratification, on-target effect monitoring, and safer, more rational development of microbiome-targeted therapies. Validated, standardized patient-reported outcome measures (PROMs) should be pre-specified as primary endpoints, alongside harmonized standard operating procedures (SOPs) for sampling and microbiome profiling, with early integration of robust safety monitoring.

CPP is best understood as a systems disorder in which microbial, immune, endocrine, and neural circuits form a tightly coupled network across the gut–vagina–endometrium–bladder axis.

Given that most women with CPP are of reproductive age, the potential impact of pelvic dysbiosis on fertility and pregnancy outcomes warrants consideration. Emerging evidence suggests that vaginal and endometrial dysbiosis may impair implantation and increase adverse perinatal risks (Moreno *et al.*, 2016; Fettweis *et al.*, 2019). Integrating reproductive outcomes into future CPP microbiome research would enhance its translational relevance.

## Conclusion

A systems perspective reframes CPP as a modifiable network, enabling more personalized care. However, evidence for microbiome-targeted interventions remains largely preclinical and human data are limited and heterogeneous. These approaches should not be considered standard of care without robust randomized trials demonstrating efficacy and safety. Given the high disease burden, rigorous evidence generation is both scientifically and ethically essential before clinical adoption.

## Supplementary Material

deag110_Supplementary_Materials_and_Methods

## Data Availability

No new data were generated or analysed in this study.

## Literature search strategy

### Search strategy and eligibility criteria

This narrative mini-review was conducted in accordance with the scope and format guidelines for mini-reviews in *Human Reproduction*. Given the narrative format of this review, no protocol pre-registration was required or undertaken. Literature searches were performed in **PubMed/MEDLINE**, supplemented by manual screening of reference lists of included articles and relevant reviews identified during the search process.

Searches were conducted between November and December 2025, with no formal lower date restriction applied. Emphasis was placed on publications from 2015 onwards to reflect the emergence of high-throughput microbiome profiling (16S rRNA amplicon sequencing and metagenomics) as standard methodology in the field. Seminal earlier works were included where judged conceptually foundational by the authors.

### Search terms

Searches combined MeSH terms and free-text keywords using Boolean operators (AND, OR, NOT). Core search strings included combinations of the following:

### Condition terms

“chronic pelvic pain”, “pelvic pain”, “endometriosis”, “adenomyosis”, “interstitial cystitis”, “bladder pain syndrome”, “irritable bowel syndrome”, “vulvodynia”, “myofascial pain syndrome”, “dysmenorrhea”

### Microbiome terms

“microbiome”, “microbiota”, “dysbiosis”, “gut microbiota”, “vaginal microbiome”, “endometrial microbiome”, “urinary microbiome”, “estrobolome”, “beta-glucuronidase”, “Lactobacillus”, “bacterial vaginosis”

### Mechanistic and metabolite terms

“short-chain fatty acids”, “butyrate”, “lipopolysaccharide”, “toll-like receptor”, “neuroinflammation”, “central sensitisation”, “gut-brain axis”, “tryptophan”, “bile acids”, “intestinal permeability”, “epithelial barrier”,

“nociception”, “pain sensitization”, “visceral hypersensitivity”, “pain modulation”, “microbial metabolites”, “metabolomics”, “multi-omics”

### Therapeutic terms

“probiotics”, “prebiotics”, “postbiotics”, “psychobiotics”, “fecal microbiota transplantation”, “vaginal microbiota transplantation”, “dietary intervention”, “low-FODMAP”

## Eligibility criteria

### Included

Original research articles (clinical observational studies, randomized controlled trials, and experimental/preclinical studies); systematic reviews and meta-analyses; narrative reviews providing mechanistic or clinical synthesis; preprints from established repositories (bioRxiv, medRxiv) when considered to provide unique and timely mechanistic evidence, clearly identified as such in the manuscript.

### Excluded

Case reports and case series; conference abstracts without accompanying full-text publications; studies not published in English; studies focused exclusively on male pelvic pain or on non-human organisms without direct relevance to the mechanistic framework discussed.

### Study selection and synthesis

Given the narrative format of this review, no formal quantitative synthesis or meta-analysis was performed. Article selection was guided by relevance to the review’s conceptual framework, methodological quality, and recency. Priority was given to multi-compartment studies, experimental models providing mechanistic evidence, and clinical studies incorporating pain outcome data. Where evidence was sparse or preliminary (e.g. virome, mycobiome, urinary microbiome in the context of CPP), this is explicitly acknowledged in the text.

All statements of causality are grounded in experimental manipulation data (e.g. antibiotic depletion, microbiota transplantation, germ-free models); observational findings are described as associations throughout the manuscript.
