# Supplementary material for: Microbiome and chronic pelvic pain in women: a mini-review
Source: Hum Reprod. 2026 Jul 24;41(9):1482–96. doi: 10.1093/humrep/deag110 (PMC13550672; doi:10.1093/humrep/deag110)
Supplement: Supplementary file 1 [file deag110_supplementary_materials_and_methods.pdf]

Literature search strategy

Search strategy and eligibility criteria

This narrative mini-review was conducted in accordance with the scope and format guidelines for mini-reviews in *Human Reproduction*. Given the narrative format of this review, no protocol pre-registration was required or undertaken. Literature searches were performed in **PubMed/MEDLINE**, supplemented by manual screening of reference lists of included articles and relevant reviews identified during the search process.

Searches were conducted between November and December 2025, with no formal lower date restriction applied. Emphasis was placed on publications from 2015 onwards to reflect the emergence of high-throughput microbiome profiling (16S rRNA amplicon sequencing and metagenomics) as standard methodology in the field. Seminal earlier works were included where judged conceptually foundational by the authors.

Search terms

Searches combined MeSH terms and free-text keywords using Boolean operators (AND, OR, NOT). Core search strings included combinations of the following:

Condition terms

“chronic pelvic pain”, “pelvic pain”, “endometriosis”, “adenomyosis”, “interstitial cystitis”, “bladder pain syndrome”, “irritable bowel syndrome”, “vulvodynia”, “myofascial pain syndrome”, “dysmenorrhea”

Microbiome terms

“microbiome”, “microbiota”, “dysbiosis”, “gut microbiota”, “vaginal microbiome”, “endometrial microbiome”, “urinary microbiome”, “estrobolome”, “beta-glucuronidase”, “Lactobacillus”, “bacterial vaginosis”

Mechanistic and metabolite terms

“short-chain fatty acids”, “butyrate”, “lipopolysaccharide”, “toll-like receptor”, “neuroinflammation”, “central sensitisation”, “gut-brain axis”, “tryptophan”, “bile acids”, “intestinal permeability”, “epithelial barrier”, “nociception”, “pain sensitization”, “visceral hypersensitivity”, “pain modulation”, “microbial metabolites”, “metabolomics”, “multi-omics”

Therapeutic terms

“probiotics”, “prebiotics”, “postbiotics”, “psychobiotics”, “fecal microbiota transplantation”, “vaginal microbiota transplantation”, “dietary intervention”, “low-FODMAP”

Eligibility criteria

Included

Original research articles (clinical observational studies, randomized controlled trials, and experimental/preclinical studies); systematic reviews and meta-analyses; narrative reviews providing mechanistic or clinical synthesis; preprints from established repositories (bioRxiv, medRxiv) when considered to provide unique and timely mechanistic evidence, clearly identified as such in the manuscript.

Excluded

Case reports and case series; conference abstracts without accompanying full-text publications; studies not published in English; studies focused exclusively on male pelvic pain or on non-human organisms without direct relevance to the mechanistic framework discussed.

Study selection and synthesis

Given the narrative format of this review, no formal quantitative synthesis or meta-analysis was performed. Article selection was guided by relevance to the review’s conceptual framework, methodological quality, and recency. Priority was given to multi-compartment studies, experimental models providing mechanistic evidence, and clinical studies incorporating pain outcome data. Where evidence was sparse or preliminary (e.g. virome, mycobiome, urinary microbiome in the context of CPP), this is explicitly acknowledged in the text.

All statements of causality are grounded in experimental manipulation data (e.g. antibiotic depletion, microbiota transplantation, germ-free models); observational findings are described as associations throughout the manuscript.
